# Epistasis, physical capacity-related genes and exceptional longevity: *FNDC5* gene interactions with candidate genes *FOXOA3* and *APOE*

**DOI:** 10.1186/s12864-017-4194-4

**Published:** 2017-11-14

**Authors:** Noriyuki Fuku, Roberto Díaz-Peña, Yasumichi Arai, Yukiko Abe, Hirofumi Zempo, Hisashi Naito, Haruka Murakami, Motohiko Miyachi, Carlos Spuch, José A. Serra-Rexach, Enzo Emanuele, Nobuyoshi Hirose, Alejandro Lucia

**Affiliations:** 1 Graduate School of Health and Sports Science, Juntendo University, Chiba, Japan; 2 Hospital Universitari Institut Pere Mata, IISPV, URV. CIBERSAM, Reus, Spain; 3 Facultad de Ciencias de la Salud, Universidad Autónoma de Chile, Talca, Chile; 4 Center for Supercentenarian Medical Research, Keio University School of Medicine, Tokyo, Japan; 5 Department of Physical Activity Research; National Institutes of Biomedical Innovation, Health and Nutrition, Tokyo, Japan; 6 Neurology Group, Galicia Sur Health Research Institute (IIS Galicia Sur), Centro de investigación biomédica en red del área de salud mental (CIBERSAM), Vigo, Spain; 7 2E Science, Robbio, (PV) Italy; 8 Centro de investigación biomédica en Envejecimiento y Fragilidad (CIBERFES), Madrid, Spain; 9 European University and Research Institute i+12, Madrid, Spain

**Keywords:** Exceptional longevity, Centenarians, FOXO3A, FNDC5, APOE, Ageing

## Abstract

**Background:**

Forkhead box O3A (*FOXOA3)* and apolipoprotein E (*APOE*) are arguably the strongest gene candidates to influence human exceptional longevity (EL, i.e., being a centenarian), but inconsistency exists among cohorts. Epistasis, defined as the effect of one locus being dependent on the presence of ‘modifier genes’, may contribute to explain the missing heritability of complex phenotypes such as EL. We assessed the potential association of epistasis among candidate polymorphisms related to physical capacity, as well as antioxidant defense and cardiometabolic traits, and EL in the Japanese population. A total of 1565 individuals were studied, subdivided into 822 middle-aged controls and 743 centenarians.

**Results:**

We found a *FOXOA3* rs2802292 T-allele-dependent association of fibronectin type III domain-containing 5 (*FDNC5*) rs16835198 with EL: the frequency of carriers of the *FOXOA3* rs2802292 T-allele among individuals with the rs16835198 GG genotype was significantly higher in cases than in controls (*P* < 0.05). On the other hand, among non-carriers of the *APOE* ‘risk’ ε4-allele, the frequency of the *FDNC5* rs16835198 G-allele was higher in cases than in controls (48.4% vs. 43.6%, *P* < 0.05). Among carriers of the ‘non-risk’ *APOE* ε2-allele, the frequency of the rs16835198 G-allele was higher in cases than in controls (49% vs. 37.3%, *P* < 0.05).

**Conclusions:**

The association of *FDNC5* rs16835198 with EL seems to depend on the presence of the *FOXOA3* rs2802292 T-allele and we report a novel association between *FNDC5* rs16835198 stratified by the presence of the *APOE* ε2/ε4-allele and EL. More research on ‘gene*gene’ and ‘gene*environment’ effects is needed in the field of EL.

## Background

Exceptional longevity (EL), defined as reaching 100+ years of age, is a generally accepted model of healthy aging with major age-related diseases usually delayed [1], and sometimes even avoided, in centenarians [2]. Age at death during adulthood has a heritability of ~ 25% [3] and consequently EL is likely to be, at least in part, a heritable phenotype. However, genome wide association studies (GWAS) of individuals with EL have mostly yielded poor results, with single nucleotide polymorphisms (SNPs) in the apolipoprotein E (*APOE*) and/or forkhead box O3A (*FOXOA3*) genes being usually the only variants achieving genome-wide significance [3], although there is no unanimity for *FOXOA3* [4]. The *APOE* ε4-allele, which is linked to a higher risk of cardiovascular and Alzheimer’s disease [5, 6], is associated with a lower likelihood of reaching EL, whereas the ε2-allele seems to show a positive association with EL and is more frequently found in centenarians than in younger controls [7]. Nonetheless, the aforementioned associations can vary by population [7]. Similarly, there is evidence that genetic polymorphisms in the *FOXO3A* gene can be associated with EL, especially the rs2802292 SNP [8–12], but such an association has not been corroborated in some cohorts [13, 14]. On the other hand, aging is inevitably associated with a decline physical function, especially in muscle mass and function (i.e., sarcopenia), with an acceleration of this process increasing the risk of mortality [15]. As such, there is a rationale for postulating the potential influence on EL of several variations in genes that are candidates to influence physical capacity and muscle function; however, up to date such candidate genes [e.g., α-actinin-3 (*ACTN3*), thyrotropin-releasing hormone (*TRHR*) or acyl-CoA synthetase long-chain family member 1 (*ACSL1*)], have not shown individual associations with EL [16, 17].

Epistasis, defined as the effect of one locus on a given phenotype being dependent on the presence of one or more ‘modifier genes’, may contribute to explain the missing heritability of complex phenotypes [18]. Though research on the role of epistasis in EL has been rather limited in the past [19], evidence for an impact of genetic interactions in determining human longevity is quickly growing. For instance, longevity may be partly determined by epistatic interactions involving mitochondrial DNA (mtDNA) loci [20]. Also, a combination of functional SNPs within adenosine deaminase (*ADA*) and tumor necrosis factor alpha (*TNF-α*) genes can influence life-expectancy in a gender-specific manner [21]. More recently, Tan et al. reported that interactions of SNPs in the *FOXO* gene family influences longevity [22]. To our knowledge, only two studies have included the *APOE* gene for evaluating gene x gene effects on longevity [23, 24]. Jazwinski et al. reported that the GTPase HRAS (also known as transforming protein p21, *HRAS1*) and ceramide synthase 1 (also known as LAG1, longevity assurance homolog, *LASS1*) genes interact with the *APOE* gene to reduce age-related increases in lipotoxic events, and haplotypes conformed by the two genes are associated with EL [23]. In the other study, epistasis analysis was performed between *APOE* haplotypes and haptoglobulin gene (*HP*) functional polymorphisms, with *HP* *1/*1 genotype protecting *APOE* ε4-carriers from age-related negative selection; in light of these results, the authors called for further research on *APOE/HP* interactions in age-related diseases such as Alzheimer’s and Parkinson’s disease [24].

Japan has the longest life expectancy in the world, as well as the highest number of individuals reaching EL [25]. Thus, Japanese long-lived people represent an attractive model to study potential genetic contributors to EL. The purpose of the present study was to assess whether the interaction between variants in genes that are candidates to influence physical capacity and energy metabolism [*ACTN3*, *TRHR*, *ACSL1*], interact with *APOE* and *FOXO3A* genes, and potentially associate with EL in the Japanese population. We also studied the potential interaction with candidate genes involved in antioxidant defense [glutathione peroxidase 1 (*GPX1*), superoxide dismutase 2 (*SOD2*)] and cardiometabolic traits [fibronectin type III domain-containing 5 (*FNDC5*), cyclin-dependent kinase inhibitor 2B antisense noncoding RNA (*CDKN2B-AS1*)],

## Results

The observed genotypic distributions of SNPs were consistent with Hardy–Weinberg Equilibrium (HWE) expectations both in controls and centenarians (*P* > 0.05). Table 1 shows the single marker allelic association results of the 12 SNPs included in the study. The frequency of the *APOE* ε2-allele was higher in cases than in controls, whereas ε4-allele frequency was lower in the former (all *P* < 0.01), as shown previously by us using part of the present cohort [26]. None of the other SNPs, including *FOXOA3* rs2802292, was associated with EL, and genotypes did not differ significantly between cases and controls in the recessive, dominant or additive models (all *P* > 0.05) (data not shown). No significant differences were observed in sex-based analyses (data not shown).

**Table 1:** Single marker allelic association results of the 12 genetic variants included in the study

Chromosome	SNP	Gene	Position	Minor allele nucleotide	Minor allele frequency		P_BONF_	OR	lower 95%CI	Upper 95%CI
					Centenarians	Controls				
1	rs16835198	*FNDC5*	32,861,080	G	0.48	0.45	0.488	1.16	1.00	1.33
2	rs1050450	*GPX1*	49,357,401	T	0.08	0.08	1	0.95	0.73	1.25
4	rs6552828	*ASCL1*	1.85E + 08	G	0.40	0.41	1	0.95	0.82	1.10
6	rs4880	*SOD2*	1.6E + 08	G	0.13	0.14	1	0.94	0.77	1.16
8	rs7832552	*TRHR*	1.09E + 08	T	0.50	0.50	1	0.97	0.85	1.12
8	rs7460	*PTK2*	1.41E + 08	T	0.36	0.36	1	1.00	0.860	1.15
8	rs7843014	*PTK2*	1.41E + 08	A	0.25	0.27	1	0.88	0.75	1.04
9	rs1333049	*CDKN2B-AS1*	22,125,504	C	0.47	0.46	1	1.05	0.91	1.21
9	rs2802292	*FOXO3A*	108,587,315	G	0.29	0.28	1	1.05	0.90	1.23
11	rs1815739	*ACTN3*	66,560,624	C	0.46	0.47	1	0.96	0.84	1.11
19	rs429358	*APOE*	44,908,684	C	0.05	0.11	6.19 × 10^−10^	0.39	0.29	0.52
19	rs7412	*APOE*	44,908,882	T	0.07	0.04	0.002	1.80	1.32	2.46

Allele frequencies were compared using chi-square statistics; ORs and 95% confidence interval (95% CI) were calculated using Plink software

*Abbreviations*: *95%CI* 95% confidence interval, *OR* odds ratio, *P*_*BONF*_ *P*-value after Bonferroni correction, *SNP* single nucleotide polymorphism

With regards to epistasis, we found a *FOXOA3* rs2802292 T-allele-dependent association of fibronectin type III domain-containing 5 (*FDNC5*) rs16835198 with EL, that is, among individuals with the rs16835198 GG genotype, the frequency of carriers of the *FOXOA3* rs2802292 T-allele was significantly higher in cases than in controls (*P* < 0.05; Table 2). On the other hand, among non-carriers of the *APOE* ‘risk’ ε4-allele, the frequency of the *FDNC5* rs16835198 G-allele was higher in cases than in controls (48.4% vs. 43.6%, *P* < 0.05; Table 3). In turn, among carriers of the ‘non-risk’ *APOE* ε2-allele, the frequency of the rs16835198 G-allele was higher in cases than in controls (49% vs. 37.3%, *P* < 0.05). No other significant gene interaction was found to be associated with EL.

**Table 2:** Genotypic frequency distribution of *FNDC5* rs16835198 according to the *FDNC5* rs16835198 T-allele presence

*FDNC5* (rs16835198) Genotype	*FOXO3A* Presence of rs2802292 T-allele		*P*-value	OR (95% CI)
	Controls (*n* = 754)	Centenarians (*n* = 668)		
GG	147 (19.5%)	162 (24.3%)	0.035	1.32 (1.03–1.70)
GT	378 (50.1%)	323 (48.3%)	NS	–
TT	229 (30.4%)	183 (27.4%)	NS	–

*Abbreviations*: *95%CI* 95% confidence interval, *NS* not significant, *OR* odds ratio

**Table 3:** Allele frequency distribution of *FNDC5* rs16835198 alleles according to *APOE* ε2/ε4 status in the study population

*FNDC5* (rs16835198)allele		Absence of *APOE* ε4-allele^a^		*Presence of APOE* ε4-allele^a^	
		Controls (2n = 1306)	Centenarians (2n = 1340)	Controls (2n = 336)	Centenarians (2n = 128)
G	N	570	648	165	62
	%	43.6%	48.4%	49.1%	48.4%
	*P*-value	0.0167		NS	
	OR (95% CI)	1.21 (1.04, 1.41)		–	
T	N	736	692	171	66
	%	56.4%	51.6%	50.9%	51.6%
	*P*-value	0.0167		NS	
	OR (95% CI)	0.83 (0.71–0.96)		–	
*FNDC5* (rs16835198)allele		Absence of *APOE* ε2-allele^a^		Presence of *APOE* ε2-allele^a^	
		Controls (2n = 1508)	Centenarians (2n = 1258)	Controls (2n = 134)	Centenarians (2n = 210)
G	n	685	607	50	103
	%	45.4%	48.3%	37.3%	49%
	*P*-value	NS		0.035	
	OR (95% CI)	–		1.62 (1.04–2.52)	
T	n	823	651	84	107
	%	54.6%	51.7%	62.7%	51%
	*P*-value	NS		0.035	
	OR (95% CI)	–		0.62 (0.40–0.96)	

*Abbreviations*: *95%CI* 95% confidence interval, *NS* not significant, *OR* odds ratio

^a^All individuals underwent genotyping for rs429358 and rs7412. The 3 major isoforms of human APOE gene (E2, E3, and E4) coded by 3 alleles (ε2, ε3, and ε4) differ in amino acid sequence at 2 sites, residue 112 (rs429358) and residue 158 (rs7412)

## Discussion

Excluding *APOE*, the present findings do not show replicable association of individual candidate genes, included those related to physical capacity and muscle function, with EL. However, we also assessed the effects of interactions among candidate polymorphisms on the one hand, and EL, on the other hand. In this regard, we report an association of *FDNC5* rs16835198 with EL that seems to depend on the presence of the *FOXOA3* rs2802292 T-allele. Moreover, we found a novel association between *FNDC5* rs16835198 stratified by the presence of the *APOE* ε2/ε4-allele, and EL. We believe this is an interesting finding because *FNDC5* gene encodes the precursor of irisin which, although there is controversy [27], was recently identified a myokine -that is, a molecule released by muscles [28]. Irisin induces expression of uncoupling protein 1 and other brown adipose tissue-associated genes [partly via increased peroxisome proliferators-activated receptor α (PPAR-α)] in white adipocytes, and thus increases thermogenesis and switching of these cells towards a brown fat-like phenotype [28]. In fact irisin has been postulated as a therapeutic agent against cardiometabolic disorders and a major component of the ‘exercise polypill’ [29].

Several mechanisms may link genetic variations in *FOXO3A* and *FNDC5* genes with EL. For example, dysregulation of the nutrient-sensing somatotropic axis [comprising growth hormone and its secondary mediator, insulin-like growth factor-1 (IGF-1)] is a major hallmark of human aging [30]. IGF-1 and insulin signaling collectively represent the “insulin and IGF-1 signaling” pathway. Among the multiple targets of this pathway is the FOXO family of transcription factors, which are also involved in aging and show striking evolutionary conservation [31]. High serum irisin levels may contribute to successful aging, with circulating levels of this biomarker being in fact significantly higher in disease-free centenarians than in young healthy controls and being particularly higher than in young patients with acute myocardial infarction [32]. Moreover, genetic variants in the *FNDC5* gene have been associated with in vivo insulin sensitivity [33], and aging is associated with alterations in insulin sensitivity/signaling [34]. Irisin might also play a significant role in reducing the risk of obesity or several related diseases [35]. In a previous report, we found no significant association between genotype and allele frequencies of polymorphisms in *FNDC5* and EL [36]. However, the rs16835198 G-allele was associated with a trend towards lower luciferase gene repoter activity in vitro [36].

Insulin sensitivity could be a link explaining the association of the combination of the two loci in *FOXO3A* and *FNDC5,* as well as in *APOE* and *FNDC5*, with EL. Insulin metabolism as well as insulin-altering therapies in Alzheimer’s disease are modulated by *APOE* status [37, 38], while irisin reduces diet-induced obesity and insulin resistance in vivo [28]. The *FNDC5* rs16835198 G-allele could favor longevity in combination with the *APOE* ε2-variant, contributing to situations in which rs16835198 SNP distribution is found among long-lived subjects in proportions similar to those found in younger controls. Thus, the conditional effects of genes on the phenotype of aging could play a role in longevity. Most studies show a lower frequency of the *APOE* ε4-allele in centenarians than in younger controls, while the *APOE* ε2-allele might be more frequent in centenarians than in younger controls [7]. A “gene x gene” interaction would introduce a modifying allele in another locus, changing the impact of the risk/protective allele from deleterious to beneficial, or vice versa. Here, we report a significant effect of the rs16835198-G allele in the *FNDC5* gene on the association between *APOE* ε2 carriage/non-carriage and EL. There are some limitations in our study. First, our results should be ideally replicated in one or more ethnically and geographically-independent cohorts to account for potential differences in ‘gene x environment’ interactions. Another drawback of case: control designs as the present one is the choice of an appropriate control group, with demographic factors, notably differences in year of birth between centenarians and controls, being potential confounders (e.g., the centenarians and controls of our study were born in the early 1900s and after 1930, respectively) [39]. Differences in gender distribution between cases and controls are also to be accounted for because genetic factors influence survival at advanced age in a sex-specific manner [40]. In this regard, the potential demographic biases of cross-sectional comparisons of genotype/allele frequencies between controls and long lived individuals could be overcome by adding more complete demographic information to genetic data, allowing for estimation of survival rates related to candidate genes [40–42]. Because we have only analyzed one SNP within the *FOXO3A* and *FNDC5* genes, further research with different variants of this or other genes involved in insulin-IGF-1, and insulin signaling collectively representing the “insulin and IGF-1 signaling” pathway, must be undertaken. On the other hand, the need for using independent populations for replication may generate some controversy. Some variants may have opposing consequences on the phenotype of interest, and the effect of gene x gene interactions on human health and lifespan might depend on several factors (e.g., internal and external exposure, including medication or different genetic backgrounds of individuals comprising the populations).

## Conclusion

We found an association between *FDNC5* rs16835198 stratified by the presence of the *FOXOA3* rs2802292 T-allele, and EL, as well as between *FNDC5* rs16835198 stratified by the presence of the *APOE* alleles, and EL. Our results suggest the need for further investigation on the possible influence of *FOXO3A/FNDC5* interactions and *APOE/FNDC5* interactions with EL, but also with other age-related diseases such as atherosclerosis, Alzheimer’s, or Parkinson’s disease. Moreover, identification of gene x gene and gene x environmental factors effects on lifespan may significantly improve our understanding of the association between genetic and non-genetic regulators of aging.

## Methods

### Study population

A total of 1565 individuals were recruited: 822 controls (aged 23–65 years; 604 women, 218 men) and 743 cases (centenarians aged 100–115 years; 623 women, 1120 men). The controls were enrolled from the Nutrition and EXercise Intervention Study (NEXIS) registered on ClinicalTrials.gov (Identifier: NCT 00926744). Inclusion criteria for the control group were being a man or woman aged 23–65 years with no history of stroke, cardiovascular disease, chronic renal failure, or walking difficulties related to knee or back pain [43]. Cases were collected from two cohorts that have been described in detail elsewhere [44]: the Tokyo Centenarians Study (TCS) and the Semi-Supercentenarians Study in Japan (SSC-J). The prevalence rates of hypertension, coronary artery disease and dementia in the Japanese centenarians were 63.6, 28.8 and 59.4%, respectively [26].

### Genotyping

Total DNA was extracted from venous blood with the QIAamp DNA Blood Maxi Kit (Qiagen, Hilden, Germany). SNP genotyping was performed using the following TaqMan® genotyping assays (Applied Biosystems, Foster City, CA): C__34204885_10 for rs16835198, Custom TaMan® Assay for rs1050450, C__30469648_10 for rs6552828, C___8709053_10 for rs4880, C__29085798_10 for rs7832552, C____243385_10 for rs7460, C__11605645_10 for rs7843014, C___1754666_10 for rs1333049, C___1841568_10 for rs2802292, C____590093_1 for rs1815739, C____904973_10 for rs7412 and C___3084793_20 for rs429358. Several quality control procedures, such as repeating the genotyping on a random 10% of the samples were carried out to assure no discrepant results in samples.

### Statistical analysis

Statistical analysis of high-density SNP genotyping data was carried out as follows: allele frequencies in cases and controls were compared using chi-square statistics, and odds ratios (OR) and 95% confidence intervals (95% CI) were calculated using Plink software [45]. Bonferroni correction was applied by dividing the *P-*value by the number of SNPs tested to give the corrected *P-*value. Allele frequencies obtained for each SNP were tested for deviations from HWE expectations. The combined effects of *APOE* and *FOXO3A* alleles and SNPs studied on the risk of EL were analyzed by binary logistic regression with SPSS v.22 statistical software (IBM, Somers, NY, USA) and OR and *P* values were calculated.

## Abbreviations

ACSL1: Acyl-CoA synthetase long-chain family member 1
ACTN3: Actinin-3
ADA: Adenosine deaminase
APOE: Apolipoprotein E
CDKN2B-AS1: Cyclin-dependent kinase inhibitor 2B antisense noncoding RNA
EL: Exceptional longevity
FNDC5: Fibronectin type III domain-containing 5
FOXOA3: Forkhead box O3A
GPX1: Glutathione peroxidase 1
GWAS: Genome wide association studies
HP: Haptoglobulin gene
HWE: Hardy–Weinberg Equilibrium
IGF-1: Insulin-like growth factor-1
mtDNA: Mitochondrial DNA
NEXIS: Nutrition and EXercise Intervention Study
OR: Odds ratios
SNPs: Single nucleotide polymorphisms
SOD2: Superoxide dismutase 2
SSC-J: Semi-Supercentenarians Study in Japan
TCS: Tokyo Centenarians Study
TNF-α: Tumor necrosis factor alpha
TRHR: Thyrotropin-releasing hormone
